# Visualizing the Distribution of Lipids in Peanut Seeds by MALDI Mass Spectrometric Imaging

**DOI:** 10.3390/foods11233888

**Published:** 2022-12-01

**Authors:** Xin Wang, Yuning Chen, Yue Liu, Lei Ouyang, Ruonan Yao, Zhihui Wang, Yanping Kang, Liying Yan, Dongxin Huai, Huifang Jiang, Yong Lei, Boshou Liao

**Affiliations:** 1 Key Laboratory of Biology and Genetic Improvement of Oil Crops, Ministry of Agriculture and Rural Affairs, Oil Crops Research Institute of the Chinese Academy of Agricultural Sciences, Wuhan 430062, China; 2 State Key Laboratory of Biocatalysis and Enzyme Engineering, School of Life Sciences, Hubei University, Wuhan 430062, China

**Keywords:** peanut, mass spectrometry imaging, spatial distribution, lipid, metabolite

## Abstract

Peanut (also called groundnut, *Arachis hypogaea* L.) seeds are used for producing edible oils and functional foods, and offer a rich source of lipids, proteins and carbohydrates. However, the location of these metabolites has not yet been firmly established. In the present study, the matrix-assisted laser desorption/ionization mass spectrometric imaging (MALDI-MSI) technique was applied to investigate spatial distribution of lipids and other key components in seeds of three peanut cultivars (ZH9, KQBH, HP). A total of 103 metabolites, including 34 lipid compounds, were putatively identified by MALDI-MSI. The abundance and spatial distribution of glycerolipids (GLs) and glycerophospholipids (GPs) were compared among the three peanut cultivars. All the identified lysophosphatidylcholine (LPC), phosphatidylethanolamine (PE) and phosphatidylcholines (PCs) were distributed mainly in the inner part of seeds. The visualization of phosphatidic acids (PAs) and triacylglycerols (TGs) revealed a dramatic metabolic heterogeneity between the different tissues making up the seed. The non-homogeneous spatial distribution of metabolites appeared to be related to the different functions of particular tissue regions. These results indicated that MALDI-MSI could be useful for investigating the lipids of foodstuffs from a spatial perspective. The present study may contribute to the development of oil crops with higher oil yields, and to improvement of food processing.

## 1. Introduction

Peanut (also called groundnut, *Arachis hypogaea* L.) is one of important oilseed and cash crops widely cultivated in the world. Since peanut seeds are rich in lipids, proteins, carbohydrates and bioactive metabolites, they are used for producing edible oil and functional foods [1]. The dietary consumption of peanut oil with high oleic acid (OA, C18:1) content in diet has diverse positive effects on human health, including the lowering of cholesterol levels, a decreased risk of inflammatory diseases, and a lower impact of long-term complications in cases of type 2 diabetes [2,3]. There are various colors of peanut testa (seed coat), including pink, black, white, and multicolor. In recent years, black peanuts become more popular in market, due to their appealing color and particular health-beneficial ingredients [4,5]. For instance, the total anthocyanin content (TAC) in deep-colored (black) peanuts has been found to be much higher than in light-colored (pink, red, white) cultivars [6,7,8].

Changes of lipid species have been measured in different high-OA peanut seeds via lipidomic approaches [9]. However, these conventional techniques are not well-suited for studying the spatial distribution of metabolites. Determining their locations would not only provide useful information for food safety purposes, but also be helpful for peanut processing and crop breeding improvements [10,11]. Currently, mass spectrometry-based imaging (MSI) is favored as a tool for visualizing the location of metabolites within various kinds of tissues without requiring extraction, purification, separation, or labeling [12]. Matrix-assisted laser desorption/ionization MSI (MALDI-MSI) and desorption electrospray ionization MSI (DES-MSI) are two major MS imaging techniques [13]. So far, MALDI-MSI has been successfully applied to analyze the spatial distribution of small molecules, lipids and proteins (peptides) in crops, such as soybeans [14], *Brassica napus* seeds [15,16], and strawberry fruits [17]. In MALDI-MSI, samples are required to be coated with a matrix, such as 1,5-diaminonaphthalene (DAN) or 2,5-Dihydroxybenzoic acid (DHB). The analytes from the surface are irradiated with laser for desorption and ionization, then ionized molecules are detected by mass spectrometry to obtain spatial information of target compounds [18].

The oil contents and lipid species of peanut kernel have been comprehensively analyzed using UPLC-Q-TOF-MS and GC-MS platforms. Researchers have found significant variances in the metabolite profiles among different cultivars and developmental stages [19]. Recently, visualization of triacylglycerols and phosphatidylcholines in *Brassica napus* seeds via MALDI-MSI revealed that lipid compositions differed significantly between distinct tissue types within the seed [15,16]. However, the locations of lipids and other key metabolites in the peanut seed matrix have not been so widely researched to date. In this study, MALDI-MSI analysis was used to investigate the spatial distribution of major components in peanut seeds. Several lipid species, including glycerolipids and glycerophospholipids, were visualized in situ and compared among the three peanut cultivars (ZH9, KQBH, HP). This study provides a landscape of peanut metabolomics from a spatial perspective, and thus should be very useful for the peanut processing industry as well as for improvements in crops.

## 2. Materials and Methods

### 2.1. Reagents and Samples

Acetonitrile and methanol were purchased from Merck (Darmstadt, Germany). MilliQ water (Millipore, Bradford, PA, USA) was used in all experiments. Carboxymethyl cellulose (CMC) sodium salt, formic acid (FA), and 2,5-Dihydroxybenzoic acid (DHB) were purchased from Sigma-Aldrich (St. Louis, MO, USA). All reagents and solvents used in this study were of analytical grade.

**Figure 1:**
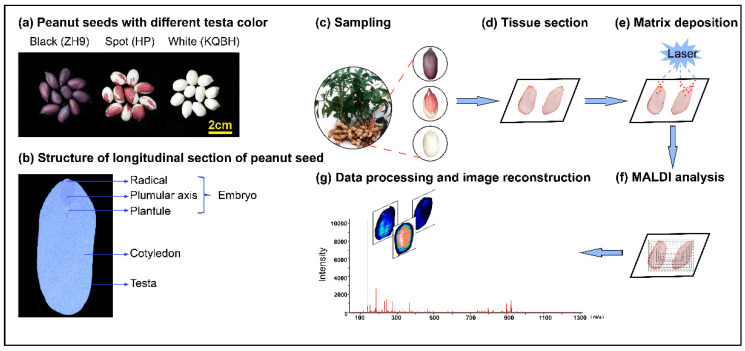
The matrix-assisted laser desorption/ionization MSI (MALDI-MSI) analysis of peanut seeds with different testa color. (**a**) Three peanut cultivars with different testa color (black, spot, and white), (**b**) structure of longitudinal section of peanut seed, and (**c**–**g**) experimental procedure of MALDI-MSI for peanut seeds.

Three peanut cultivars with different testa colors were used in this study. “Zhonghua 9” (ZH9) is a black peanut, “Kangqibaihong” (KQBH) has white testa, and “Huapi” (HP) has a red seed coat with white spot (Figure 1a). The peanut seed structure was mainly distinguished as embryo, cotyledon, and testa. From a histological point of view, the embryo was divided into three parts, including radical, plumular axis, and plantule (Figure 1b). All plant materials were grown in the test field of Oil Crop Research Institute, Chinese Academy of Agricultural Sciences, Wuhan, China. The peanuts were harvested at the mature stage (about 120 days after planting), and seeds with similar size (about 2 cm in longitudinal direction) were collected (Figure 1c). After removing testa, seeds were immersed with 10% gelatin (wt/vol) solutions in an embedding box, then frozen on dry ice and stored in a −80 °C refrigerator until use for MALDI-MSI analysis.

### 2.2. Peanut Sample Section and Spray

The typical process of MALDI-MSI is shown in Figure 1d–g. Firstly, frozen tissues were placed in the cryostat chamber to equilibrate at −20 °C for 60 min. The seed samples were sectioned longitudinally at 8 μm thickness using a Leica CM1950 cryostat (Leica Microsystems GmbH, Wetzlar, Germany) at −20 °C. The tissue sections were placed in groups on electrically conductive slides coated with indium tin oxide (ITO), and then dried in a vacuum desiccator for 30 min. After desiccation, DHB matrix solution at 15 mg/mL (in 90% acetonitrile) was sprayed onto the tissues using a TM-Sprayer (HTX Technologies LLC, Chapel Hill, NC, USA). The spraying parameters were optimized as follows: 60 °C spray nozzle temperature, 0.12 mL/min flow rate, 5 psi spray air pressure, and 12 passes with 6 s drying time for each cycle.

### 2.3. MALDI-MSI Parameters

The peanut sections were analyzed using a prototype Bruker timsTOF flex MS system (Bruker Daltonics, Bremen, Germany) equipped with a 10 kHz smart beam 3D laser (Figure 1f). The laser power was set to 80%. The data were acquired in a 50 μm step size, and *m*/*z* values in range of 50–1300 Da were measured in positive ion mode. MALDI mass spectra were normalized with the root mean square, and the signal intensity in each image was shown as the normalized intensity. For MS/MS analysis, the selected precursor ions and the product ions were obtained by the timsTOF flex MS system in the MS/MS mode. For the identification of metabolites, the obtained MS/MS spectra were compared with those of compounds in a self-built database MWDB as well as with standard MS/MS spectra in the publicly available metabolite databases [20]. If the ion score for an MS/MS match was low, the measured accurate mass of the parent ion of the compound was used for metabolome database search with a threshold error of less than 10 ppm. The highest matched and/or scored compound was then exported for further confirmation and analysis, including querying of literatures and manual spectral interpretations.

### 2.4. Data Analysis

Raw MSI data were first analyzed using FlexImaging (v4.0) software (Bruker Daltonics, Germany) and then imported to SCiLS Lab software (Bruker Daltonics, Germany) for further analysis (Figure 1g). A whole tissue section was selected as a region of interest (ROI). The ion intensity values of mass spectrum were exported from SCiLS Lab software. The average intensity/ROI area (mm^2^) was used to compare the relative abundance of respective metabolite ions. For multivariate statistical analysis, partial least squares-discriminant Analysis (PLS-DA) was used to identify the alternation of metabolites among different groups. An absolute value of log_2_ (fold change) ≥ 1 and variable importance in the projection (VIP) > 1.0 were introduced to screen significant differential metabolites (SDMs). The data were expressed as means± standard deviation for three biological replicates.

## 3. Results and Discussion

### 3.1. Identification of Differential Metabolites in Peanut Seeds by MALDI-MSI

**Figure 2:**
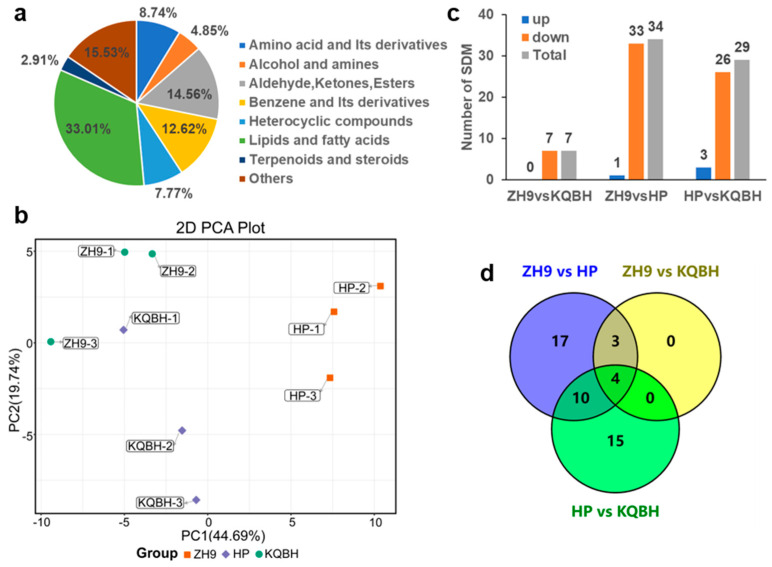
Identification of differential metabolites in the peanut seeds through MALDI-MSI. (**a**) The classification of 103 metabolites detected by MALDI-MSI, (**b**) principal component analysis (PCA) of MALDI-TOF MS mass spectra of the three peanut cultivars (three biological replicates), (**c**) summary of significant differential metabolites (SDMs) between different comparison sets, and (**d**) Venn diagram of SDMs among comparison sets.

A total of 103 metabolites were detected by MALDI-MSI (Table S1), and these were divided into eight subgroups (Figure 2a), with lipids being the largest (34 metabolites in this subgroup). Principal component analysis (PCA) showed a clear separation of peanut seeds of different cultivars except for the sample “KQBH-1” (Figure 2b). According to the metabolite abundance obtained from MALDI-MSI data, significant differential metabolites (SDMs) were screened among different peanut cultivar seeds (Figure 2c). A total of 49 SDMs were identified in the three comparison sets (ZH9 vs HP, ZH9 vs KQBH, HP vs KQBH), and most of the SDMs belonged to lipids (Figure 2d, Table S2).

### 3.2. Spatial Distribution of Glycerophospholipids

**Figure 3:**
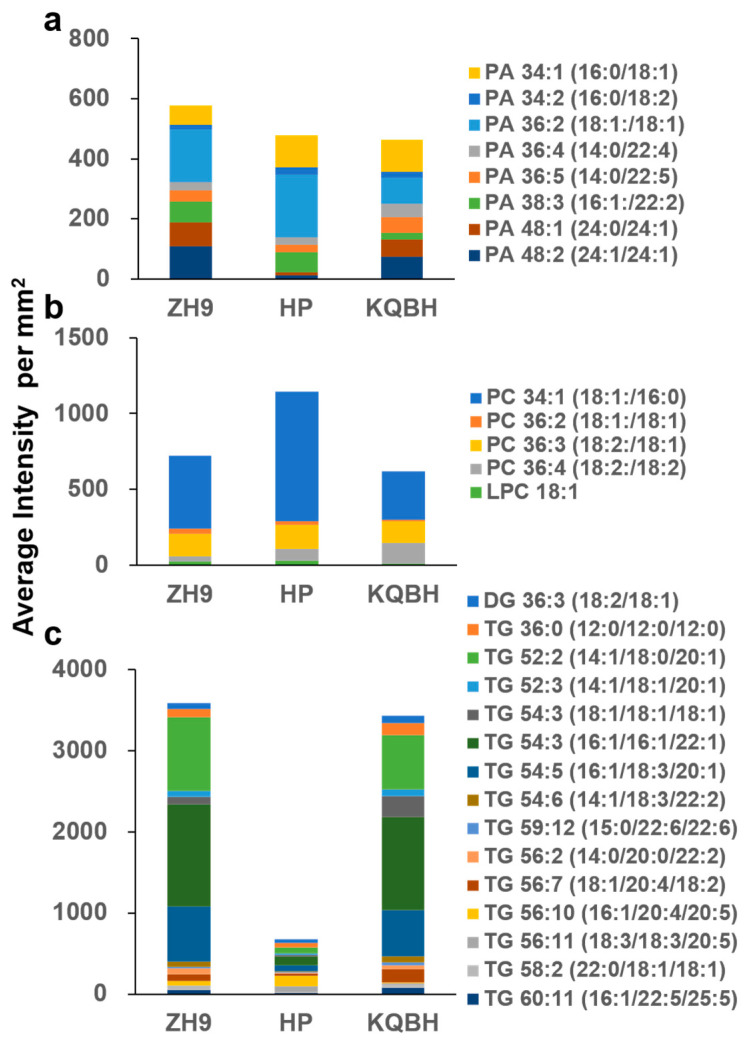
The stacked column chart showed the average intensity of (**a**) phosphatidic acids, (**b**) phosphatidylcholines, and (**c**) triacylglycerols by MALDI-MSI from different peanut seeds. A whole tissue section was selected as a region of interest. The average intensity per mm^2^ represents the relative abundance of lipid species.

**Figure 4:**
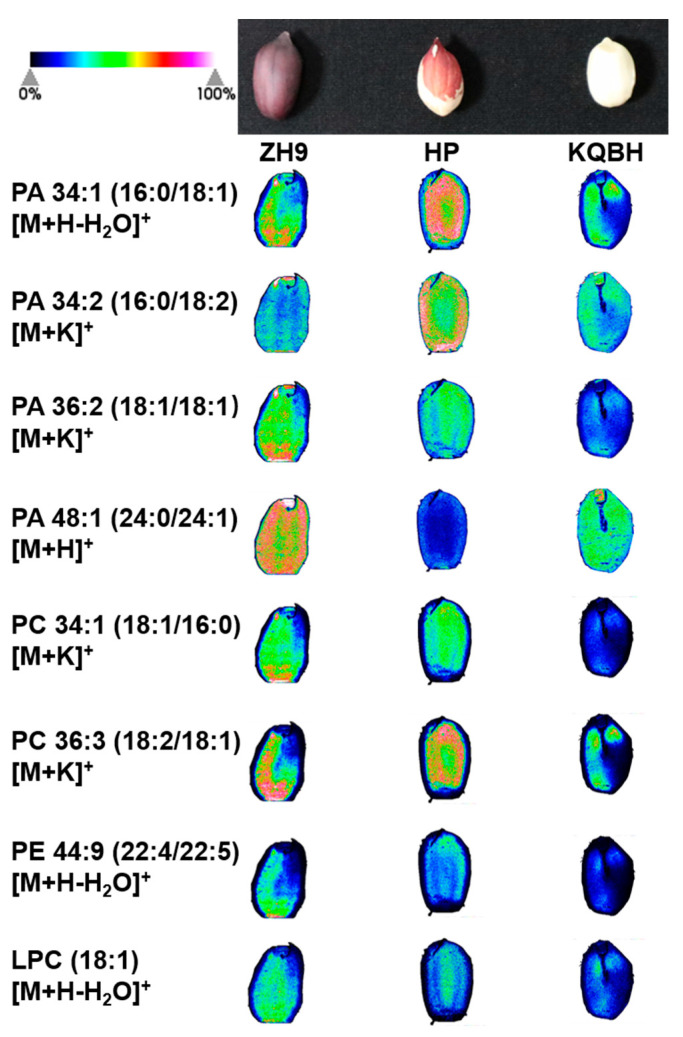
MALDI-MSI of representative glycerophospholipids in different peanut seeds. The left column shows the names of phosphatidic acids (PAs), phosphatidylcholines (PCs), lysophosphatidylcholine (LPC), and phosphatidylethanolamine (PE). The green (minimum) to red (maximum) scale indicates ion intensity for each lipid species determined by mass spectrometry. For the same glycerophospholipid molecules, signal range was normalized at the same level.

Since lipids were found to be the largest subgroup of the detected metabolites, their spatial distributions were explored by MALDI-MSI. Glycerophospholipids (GPs, also called phospholipids) are major components of cell membranes, and can be divided into different subclasses based on the nature of the polar headgroup at the terminal carbon of the glycerol backbone [21]. In total, 14 GPs were putatively identified in the three peanut cultivars, including eight phosphatidic acids (PAs), four phosphatidylcholines (PCs), one phosphatidylethanolamine (PE) and one lysophosphatidylcholine (LPC) (Table 1). PA is the first lipid species formed by complete acylation of glycerol-3-phosphate (glycerol-3-P), and is essential for several aspects of plant development and stress responses [22]. As shown by the histogram in Figure 3a, the most abundant (average intensity per mm^2^) PA species were PA 36:2 (18:1/18:1) and, to a lesser extent, PA 34:1 (16:0/18:1). These results were expected, given that oleic acid (18:1) is the most abundant fatty acid in the majority of peanut cultivars (Wang et al., 2012). The MSI results showed that these two major PAs (PA 34:1, PA 36:2) had a similar localization, involving more abundant distribution in the inner cotyledons of peanuts (Figure 4). The other PAs, such as PA 34:2 (16:0/18:2) and PA 36:5 (14:0/22:5), were accumulated in a contrary order (Figure S1). This spatial difference was more distinct for PA 34:1 (16:0/18:1) and PA 34:2 (16:0/18:2) in the HP cultivar. Additionally, PA 48:1 (24:0/24:1) was localized in the whole cotyledon of ZH9, but exhibited a notably higher level in the embryo of KQBH.

Four PCs (PC 34:1, PC 36:2, PC 36:3 and PC 36:4) were putatively identified in the samples (Figure 4, Figure S1). It was found that PC 34:1 (18:1/16:0) was the most abundant PC molecular species in the samples (Figure 3b). Interestingly, all the identified PCs were distributed mainly in the inner part of tissue slices. This was representative for PC 36:3 (18:2/18:1) in the HP peanut. LPCs are hydrolyzed GPs that are generated by removing a fatty acid acyl moiety from PCs. Only one LPC (LPC 18:1) was identified in this study. PE is frequently the second-most abundant GP subclass in plant tissues, and one PE (PE 44:9) was detected in the tissue slice. Both LPC 18:1 and PE 44:9 displayed similar localization characteristics to the PCs, but exhibited much lower intensities.

### 3.3. Spatial Distribution of Glycerolipids

**Figure 5:**
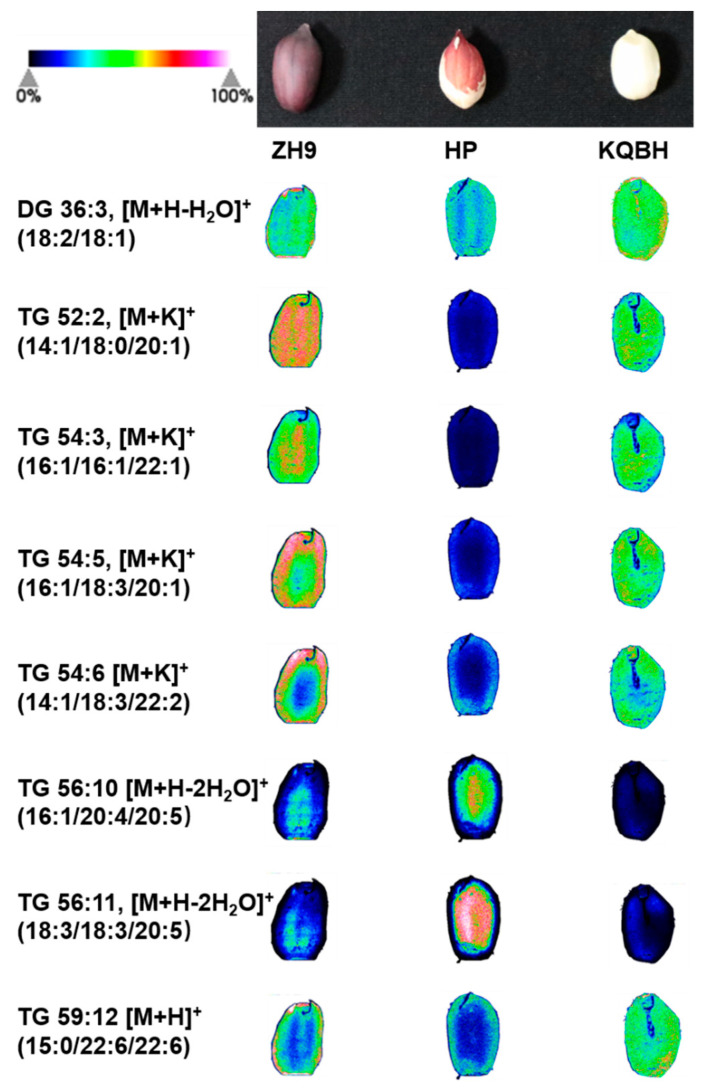
MALDI-MSI of representative glycerolipids in different peanut seeds. The left column shows the name of triacylglycerols (TGs) and diacylglycerol (DG). The green (minimum) to red (maximum) scale indicates ion intensity corresponding to each lipid species determined by mass spectrometry. For the same lipid individuals, signal range was normalized at the same level.

Glycerolipids (GLs) are the primary lipids in peanuts, with triacylglycerols (TGs) being the major form in dietary oils [23]. TG is comprised of three fatty acids esterified to a glycerol molecule. During the biosynthesis and digestion of TGs, diacylglycerols (DGs) and monoacylglycerols (MGs) are produced as intermediates, with the substitution of two fatty acids and one fatty acid, respectively, at the glycerol backbone [24]. In the present study, 15 GLs were tentatively identified, including 14 TGs and one DG. As shown in Figure 3c, the total abundance of TGs was significantly higher in ZH9 and KQBH than in HP samples. It was found that TG 54:3 (16:1/16:1/22:1) was the most abundant TG species, while some other TGs, such as TG 54:6 (14:1/18:3/22:2) and TG 59:12 (15:0/22:6/22:6), were scarcely detected. The MSI results indicated that the spatial distribution of TGs differed among different peanut cultivars (Figure 5, Figure S2). For instance, TG 52:3, TG 54:5, TG 54:6, TG 56:7, TG 59:12, and TG 60:1, were all more abundant in the outer part or edge of cotyledons. This was evident for TG 54:5 (16:1/18:3/20:1) and TG 54:6 (14:1/18:3/22:2) in ZH9. TG 56:10 (16:1/20:4/20:5) and TG 56:11 (18:3/18:3/20:5) exhibited higher levels in the inner part or center of tissue slices, especially in HP samples. The remaining six TGs and DG 36:3 (18:2/18:1) were found to be distributed across the slices, and this was prominent for TG 52:2 (14:1/18:0/20:1) in ZH9.

In summary, visualizing lipid species by MALDI-MSI indicated that many TG species were distributed heterogeneously in different peanut seed tissues. This has been commonly found in other crops, including *Gossypium hirsutum*, *Camelina sativa*, and *Brassica napus* [14,25,26,27]. In plants, multiple TG biosynthesis routes occur in particular tissues and distinct subcellular organelles. The differences in the localization of lipids might be attributed to the tissue-specific expression of structural enzymes involved in lipid metabolism. In oilseed rape, for example, the aleurone layer was found to have higher contents of TG species containing 18:2 and 18:3 compared with embryonic tissues. This might be due to substantial levels of endoplasmic reticulum-localized fatty acid desaturases in these tissue regions [15]. In this study, it was found that the inner and outer peanut cotyledons were enriched with different TGs and PCs, despite being functionally similar tissues. The molecular mechanism underlining this heterogenous distribution pattern remains largely unknown. By combining the MALDI-IMS results with spatial transcriptomics and/or proteomics data [28,29], future studies might elucidate the tissue-specific lipid biosynthesis pathway, and thereby promoter a better understanding of the spatial pattern of metabolites in peanut seeds.

### 3.4. Spatial Distribution of Other Key Metabolites

**Figure 6:**
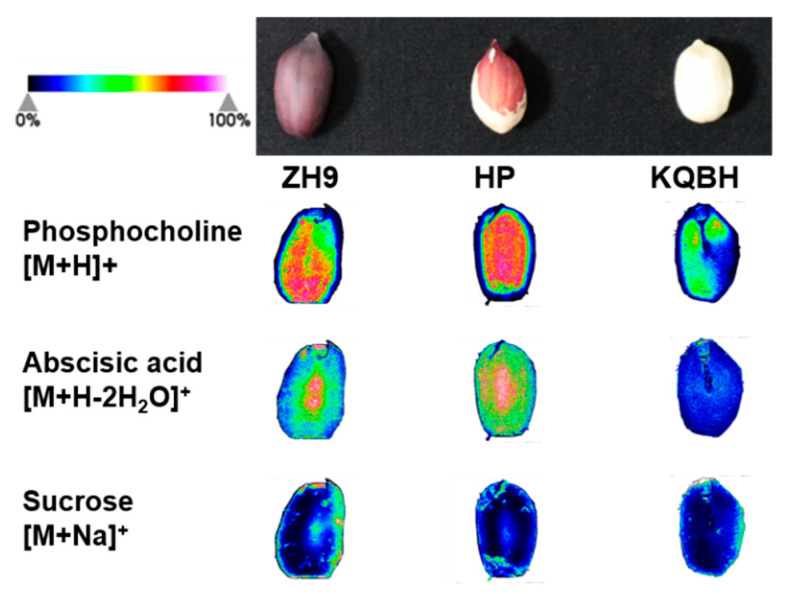
MALDI-MSI of key metabolites in different peanut seeds. The green (minimum) to red (maximum) scale indicates ion intensity corresponding to each metabolite determined by mass spectrometry.

In addition to lipid species, several key metabolites were identified and visualized in situ in peanut tissue slices including phosphocholine, which is an important component involved in phospholipid metabolism. The MALDI-MSI images showed both ZH9 and HP seeds had a strong average intensity of phosphocholine, which was distributed more abundantly in the inner part of cotyledons than in the other areas (Figure 6). The distribution pattern of phosphocholine was similar to that of PCs, which is likely due to the fact that phosphocholine is a precursor for PC biosynthesis [30]. Moreover, abscisic acid (ABA) plays a vital role in regulating embryo development and lipid metabolism in seeds [31,32]. The ZH9 and HP peanut had a great abundance of ABA which was distributed mainly in the central part of cotyledons. In contrast, the KQBH seeds had a lower level of ABA that distributed evenly throughout the whole slice. Generally, sucrose accounts for over 90% of the total sugar content in peanut kernels, and is a key factor influencing its flavor and nutritional quality [33]. The IMS results showed that sucrose was distributed throughout the whole tissue. with a slightly higher ion intensity on the edge of cotyledon in the three peanut cultivars There was no significance difference in the relative abundance of sucrose among the three peanut varieties. Similar results have been obtained in coffee bean seeds where sucrose was detected across the whole endosperm [34].

## 4. Conclusions

In the present study, the major lipid species and several key metabolites were identified and mapped in sections of peanut seed using the MALDI-MSI technique. The abundance and spatial distribution of metabolites were compared among three peanuts with different testa colors. Interestingly, all the identified PCs, LPC and PE were distributed mainly in the inner part of seeds. In contrast, visualization of TG as well as PA molecular species revealed a highly heterogenous distribution pattern within peanut seeds, which was likely to be related to the tissue-specific metabolic pathways involved in lipid biosynthesis. These results indicate that MALDI-MSI could be useful for investigating the lipids of foodstuffs from a spatial perspective. The present study may contribute to the development of oil crops with higher oil yields, and also to improvements in food processing.

## Figures and Tables

**Table 1:** The information of lipids and major compounds putatively identified by MALDI-MSI. ^a^ the measured accurate mass of the parent ion of compound, ^b^ score was obtained from the highest matched compound in a self-built database MWD and publicly available metabolite databases, and ^c^ 14 triacylglycerols (TG), 8 phosphatidic acids (PA), 4 phosphatidylcholines (PC), one diacylglycerol (DG), one lysophosphatidylcholine (LPC) and one phosphatidylethanolamine (PE) were putatively identified in the samples.

Types	*m*/*z*^a^	Score ^b^	Adduct	Formula	Putative Compound ^c^
DG	618.5223	0.98	M+H-H_2_O	C_39_H_70_O_5_	DG 36:3 (18:2/18:1)
TG	638.5485	0.97	M+H-2H_2_O	C_39_H_74_O_6_	TG 36:0 (12:0/12:0/12:0)
	896.6894	0.73	M+H-2H_2_O	C_59_H_92_O_6_	TG 56:11 (18:3/18:3/20:5)
	898.7050	0.68	M+H-2H_2_O	C_59_H_94_O_6_	TG 56:10 (16:1/20:4/20:5)
	856.7520	0.98	M+Na	C_55_H_100_O_6_	TG 52:3 (14:1/18:1/20:1)
	858.7676	0.87	M+K	C_55_H_102_O_6_	TG 52:2 (14:1/18:0/20:1)
	952.7520	0.52	M+Na+HCOOH	C_63_H_100_O_6_	TG 60:11 (16:1/22:5/25:5)
	904.7520	0.58	M+K+HCOOH	C_59_H_100_O_6_	TG 56:7 (18:1/20:4/18:2)
	884.7833	0.79	M+Na	C_57_H_104_O_6_	TG 54:3 (18:1/18:1/18:1)
	878.7363	0.89	M+K	C_57_H_98_O_6_	TG 54:6 (14:1/18:3/22:2)
	880.7520	0.96	M+K	C_57_H_100_O_6_	TG 54:5 (16:1/18:3/20:1)
	884.7833	0.98	M+K	C_57_H_104_O_6_	TG 54:3 (16:1/16:1/22:1)
	936.7207	1.00	M+H	C_62_H_96_O_6_	TG 59:12 (15:0/22:6/22:6)
	914.8302	0.99	M+K	C_59_H_110_O_6_	TG 56:2 (14:0/20:0/22:2)
	942.8615	0.98	M+K	C_61_H_114_O_6_	TG 58:2 (22:0/18:1/18:1)
PA	672.4730	0.97	M+K	C_37_H_69_O_8_P	PA 34:2 (16:0/18:2)
	694.4574	0.92	M+K	C_39_H_67_O_8_P	PA 36:5 (14:0/22:5)
	696.4730	0.95	M+K	C_39_H_69_O_8_P	PA 36:4 (14:0/22:4)
	754.4550	0.98	M+H-H_2_O	C_37_H_72_O_11_P_2_	PA 34:1 (16:0/18:1)
	700.5043	0.89	M+K	C_39_H_73_O_8_P	PA 36:2 (18:1:/18:1)
	726.5200	0.98	M+K	C_41_H_75_O_8_P	PA 38:3 (16:1:/22:2)
	868.6921	0.99	M+H	C_51_H_97_O_8_P	PA 48:2 (24:1/24:1)
	870.7078	0.99	M+H	C_51_H_99_O_8_P	PA 48:1 (24:0/24:1)
PC	759.5778	0.94	M+K	C_42_H_82_NO_8_P	PC 34:1 (18:1:/16:0)
	785.5935	0.98	M+Na	C_44_H_84_NO_8_P	PC 36:2 (18:1:/18:1)
	781.5622	0.99	M+K	C_44_H_80_NO_8_P	PC 36:4 (18:2:/18:2)
	783.5778	0.96	M+K	C_44_H_82_NO_8_P	PC 36:3 (18:2:/18:1)
PE	841.5622	0.95	M+H-H_2_O	C_49_H_80_NO_8_P	PE 44:9 (22:4/22:5)
LPC	521.3481	0.86	M+H-H_2_O	C_26_H_52_NO_7_P	LPC 18:1
Other	183.0660	0.91	M+H	C_5_H_14_NO_4_P	Phosphocholine
	264.1362	0.80	M+H-2H_2_O	C_15_H_20_O_4_	Abscisic acid
	342.1162	0.92	M+Na	C_12_H_22_O_11_	Sucrose

## Data Availability

The data presented in this study are available on request from the corresponding author.

## Supplementary Materials

The following supporting information can be downloaded at: https://www.mdpi.com/article/10.3390/foods11233888/s1, Figure S1: The mass spectrometry imaging of phosphatidic acid (PA) and phosphatidylcholine (PC) in peanut seeds with different testa color; Figure S2: The mass spectrometry imaging of triacylglycerol (TG) in peanut seeds with different testa color; Table S1: List of metabolites detected by MALDI-MSI; Table S2: Significant differential metabolites (SDMs) between different samples.
