# Supplementary material for: Visualizing the Distribution of Lipids in Peanut Seeds by MALDI Mass Spectrometric Imaging
Source: Foods. 2022 Dec 1;11(23):3888. doi: 10.3390/foods11233888 (PMC9739101; doi:10.3390/foods11233888)
Supplement: Supplementary file 1 [file foods-11-03888-s001.zip › Figure S.pdf]

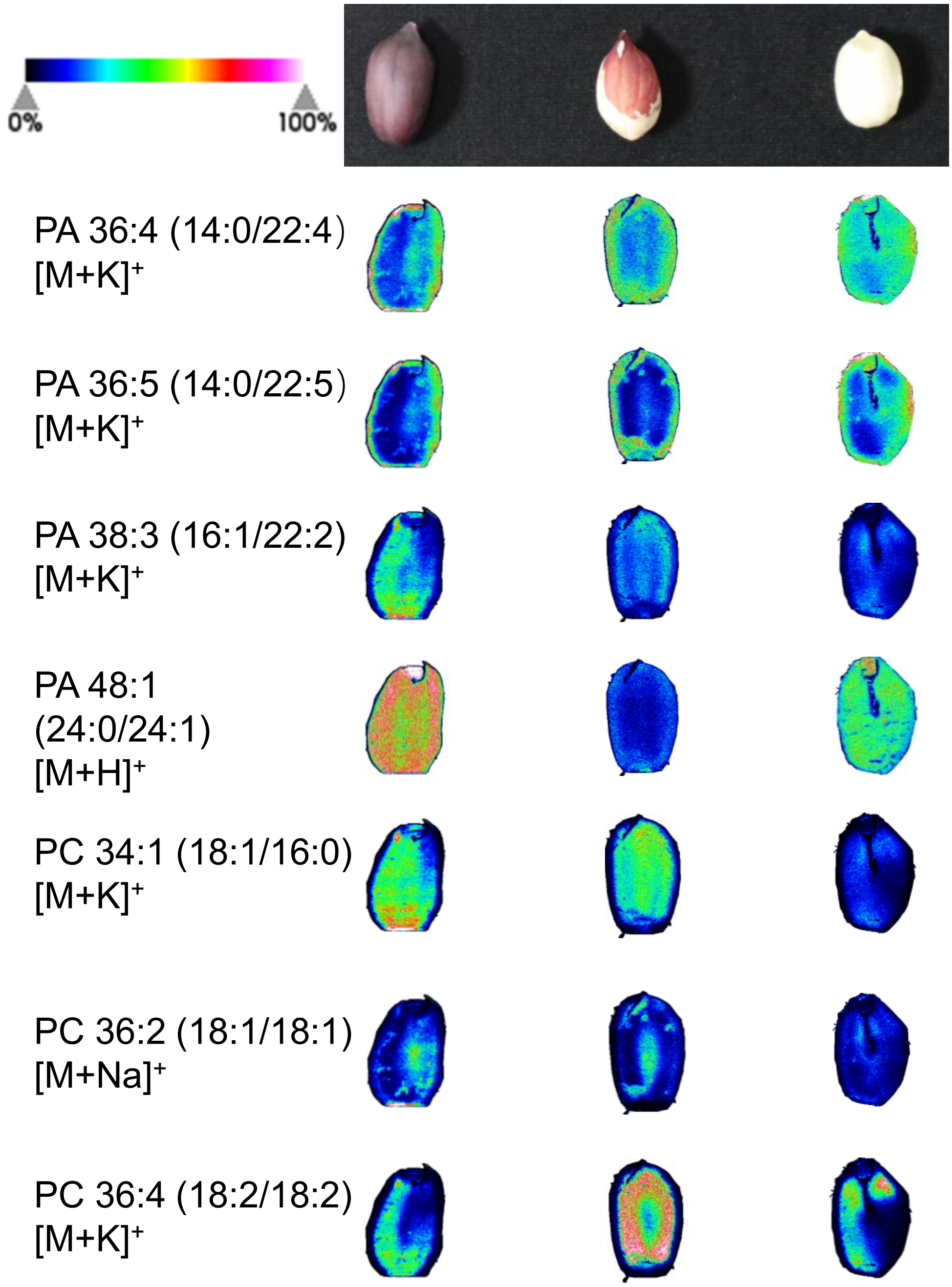

Figure S1. MALDI-MSI of of phosphatidic acid (PA) and phosphatidylcholine (PC) in peanut seeds. The first row is the photo of peanut seeds for tissue slices, and the first column is the name for PA and PC. Green (minimum) to red (maximum) scale represent ion intensity corresponding to each lipid species determined by high resolution mass spectrometry. For the same lipid species, the signal range was normalized at the same level.

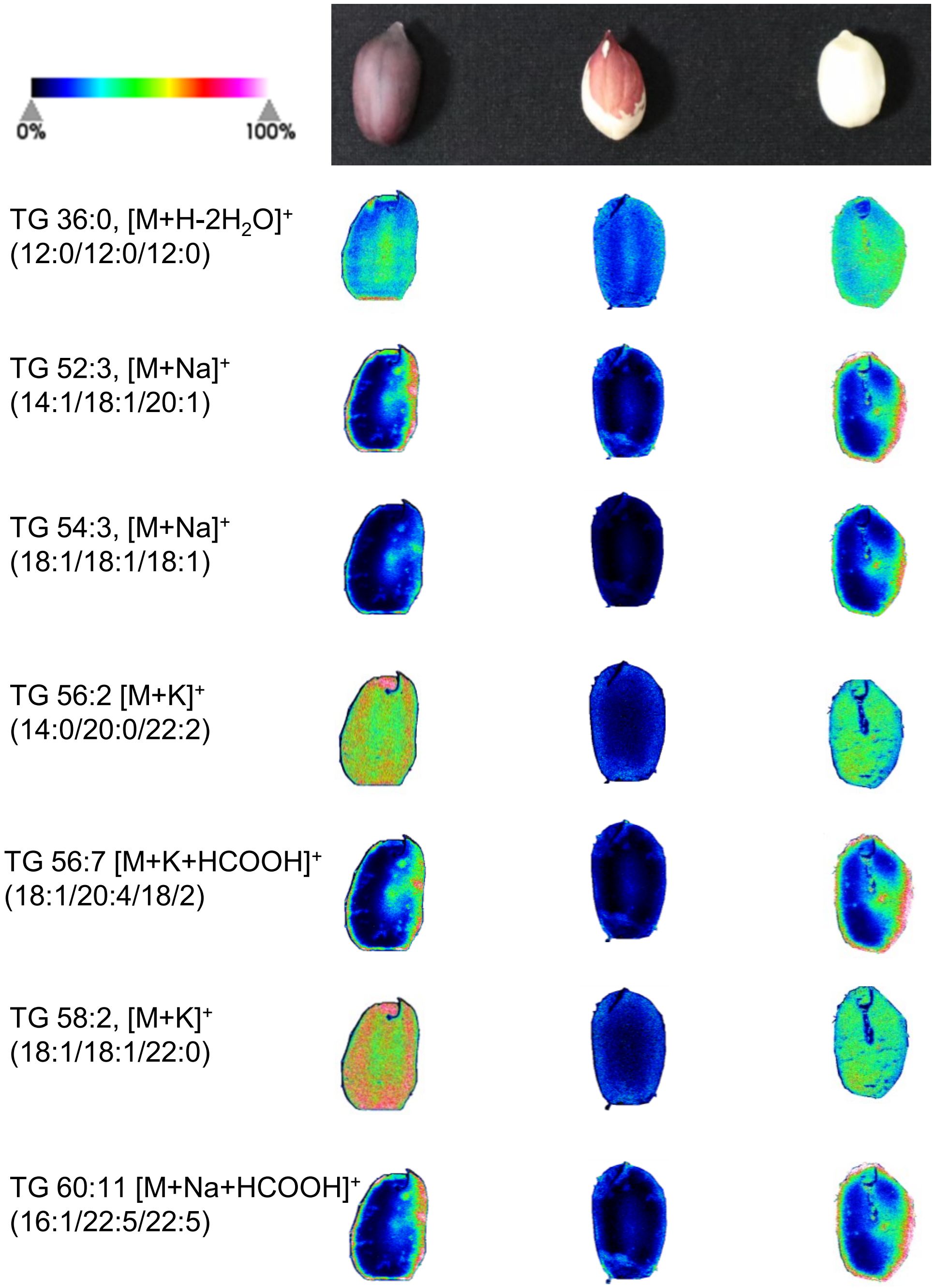

Figure S2. MALDI-MSI of of triacylglycerol (TG) in peanut seeds. The first row is the photo of peanut seeds for tissue slices, and the first column is the name for TG. Green (minimum) to red (maximum) scale represent ion intensity corresponding to each lipid species determined by high resolution mass spectrometry. For the same TG individual, the signal range was normalized at the same level.
